# Anti-sulfatide antibody-positive Guillain–Barré syndrome in adults following off-craniotomy for cerebellar contusion: A case report

**DOI:** 10.1097/MD.0000000000040970

**Published:** 2024-12-27

**Authors:** Xiaobin Min, Haoye Feng, Riguang Zhao, Zhigang Guo, Hongjun Su

**Affiliations:** a Department of Neurosurgery, Tianjin Baodi Hospital, Baodi Hospital of Tianjin Medical University, Tianjin, P.R. China; b Department of Neurology, Tianjin Baodi Hospital, Baodi Hospital of Tianjin Medical University, Tianjin, P.R. China.

**Keywords:** anti-sulfatide, cerebellar contusion, craniotomy, Guillain–Barré syndrome, immunoglobulin

## Abstract

**Rationale:**

Gullain–Barré syndrome (GBS) is a rare autoimmune condition primarily presenting with symmetrical progressive limb weakness. It is frequently associated with sensory and autonomic symptoms and autonomic disturbances and often manifests seropositivity for anti-ganglioside antibodies. Infections are considered major precipitants; however, GBS post-craniotomy for severe traumatic brain injury is a rarity.

**Patient concerns:**

A 79-year-old female underwent craniotomy for a cerebellar contusion sustained from severe traumatic brain injury, leading to quadriplegia, autonomic dysfunction, dilated pupils, and respiratory failure. However, the patient’s GBS manifested slightly differently. Her limb weakness was asymmetric and progressed from 1 upper limb to the other.

**Diagnoses:**

The diagnosis of GBS was confirmed based on clinical presentation, cerebrospinal fluid analysis showing albuminocytologic dissociation, and the detection of anti-sulfatide antibodies in serum.

**Interventions:**

The patient received intravenous immunoglobulin (IVIG) therapy at 2 g/kg daily, along with supportive measures including mechanical ventilation and rehabilitation.

**Outcomes:**

The patient demonstrated significant improvement within 5 days of IVIG treatment, achieving near-complete functional recovery with grade 4 muscle strength at discharge 6 weeks post-intervention.

**Lessons:**

This case highlights the need to consider GBS in postoperative patients with acute limb weakness, even in atypical presentations. Early recognition and timely IVIG treatment are critical for favorable outcomes.

## 1. Introduction

Guillain–Barré syndrome (GBS) is an autoimmune disease, and the risk of GBS over a person’s lifetime is estimated at 1 in 1000. It affects the nerves outside the brain and spinal cord (the peripheral nerves) and develops over several days to weeks.^[1]^ Although individuals of any age can develop GBS, the incidence increases with age, and males are slightly more likely to develop GBS than females.^[2]^ GBS can cause severe muscle weakness, and death occurs in about 5% of patients. The most common subtypes are acute inflammatory demyelinating polyradiculoneuropathy and acute motor axonal neuropathy.^[3]^ It is an acute, postinfectious immune-mediated polyradiculoneuropathy typically arising a few days to 6 weeks after bacterial or viral infections including *Campylobacter jejuni*, *Haemophilus influenzae*, *Mycoplasma pneumoniae*, influenza, Epstein–Barr virus, cytomegalovirus, and more recently, Zika virus.^[4,5]^ It is, however, often associated with positive anti-ganglioside antibodies.^[6]^ It can also be triggered by pregnancy, surgery, or even vaccination.^[7,8]^ The mechanism by which surgery leads to GBS is currently not fully understood and may be the result of a systemic inflammatory response induced by surgical stress.^[9]^ Posterior fossa craniotomy as an inciting event is very uncommon with few reported cases. We reported a case of anti-sulfatide antibody-positive GBS following off-craniotomy for cerebellar contusion.

## 2. Case presentation

### 2.1. General information

A 79-year-old woman was admitted to the neurological intensive care unit for observation due to a careless fall. After the injury, the patient immediately developed coma, accompanied by nausea and vomiting. However, the patient was somnolent and Glasgow Coma Scale score was 14 on admission (eye-opening: 3 scores; verbal response: 5 scores; motor response: 6 scores). She denied a history of any infections, hypertension, diabetes, or heart disease.

### 2.2. Admission examination

**Figure 1.**
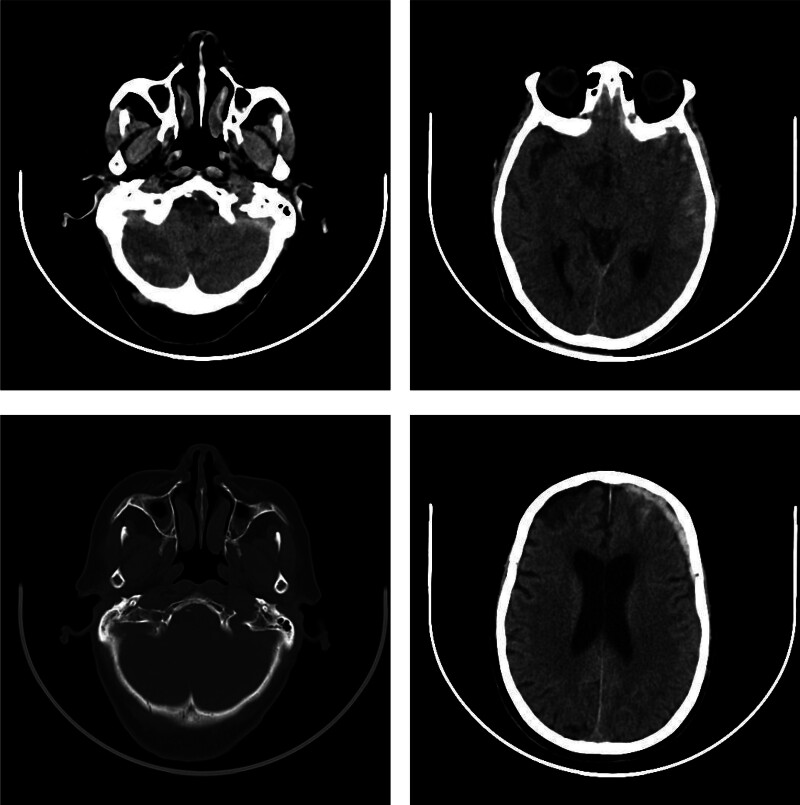
Head CT image of the patient admitted to the emergency department. CT = computed tomography.

Emergency On non-contrast head computed tomography (CT), she was found to have contusion and laceration of left frontal lobe, left temporal lobe, and right cerebellar hemisphere with attendant traumatic subarachnoid hemorrhage, subdural hematomas, and occipital fracture (Fig. 1). Electrocardiography revealed no abnormalities.

The analysis of the cerebrospinal fluid (CSF) showed a total protein of 1.3 g/L and nuclear cell count of 3/µL. This indicated the albuminocytological dissociation. Anti-ganglioside antibodies (only anti-sulfatide antibodies) were detected to be positive in the patient’s serum but not in his CSF.

### 2.3. Treatment

**Figure 2.**
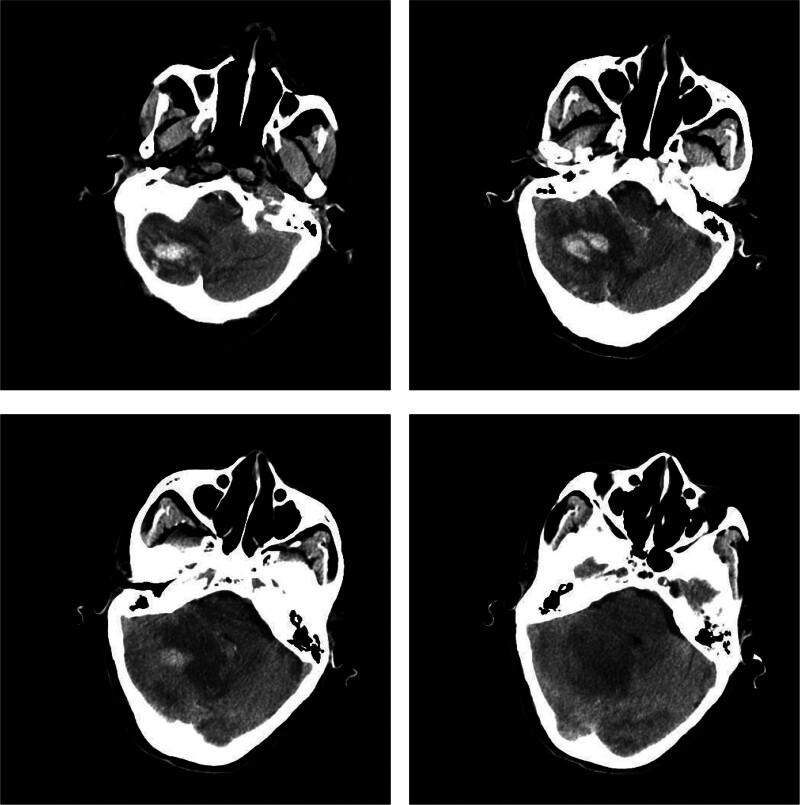
Review of head CT image before craniotomy for exacerbation on the day of admission. CT = computed tomography.

**Figure 3.**
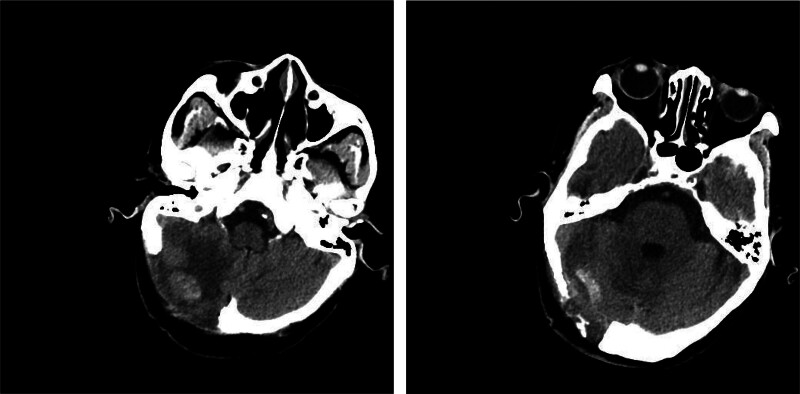
CT image of the head on the first postoperative day. CT = computed tomography.

Supportive treatments for the patient after admission were performed, such as electrocardio and blood pressure monitor, proton pump inhibitors to inhibit gastric acid secretion, neurotrophic drugs, and regular craniocerebral CT scans, but without gangliosides or their derivatives. Six hours after admission, the patient’s condition became worse, and her consciousness state was drowsy. An emergency head CT (Fig. 2) showed no new intracranial rebleeding. However the cerebellar hemisphere contusion and laceration were aggravated, accompanied by a local area of low-density edema was observed around the hematoma. At the same time, the patient’s brain stem was compressed and the cisterns were not clearly displayed. Therefore, cerebellar contusion and hematoma evacuation, along with decompressive craniectomy, were performed. On the first postoperative day, the patient underwent a follow-up cranial CT scan. The results of Figure 3 demonstrated post-evacuation alterations of a contusion hemorrhage in the right cerebellar hemisphere, with the creation of a local bone window. The interpeduncular and perimesencephalic cisterns were patent, and the previously observed brainstem compression was relieved. The patient’s symptoms continued to improve after surgery, and there was no motor and sensory disturbance.

### 2.4. Diagnosis of GBS

Seven days after surgery, the patient’s condition suddenly worsened, showing weakness in the right limb, especially in the upper limb. Ten hours later, the disease progressed to quadriplegia, autonomic dysfunction, dilated pupils, and respiratory failure. She was treated with endotracheal intubation and mechanical ventilation, but conscious.

**Figure 4.**
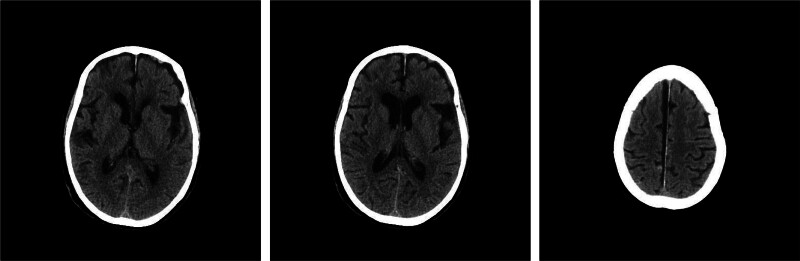
Repeat head CT image after consideration of Guillain–Barré syndrome. CT = computed tomography.

**Figure 5.**
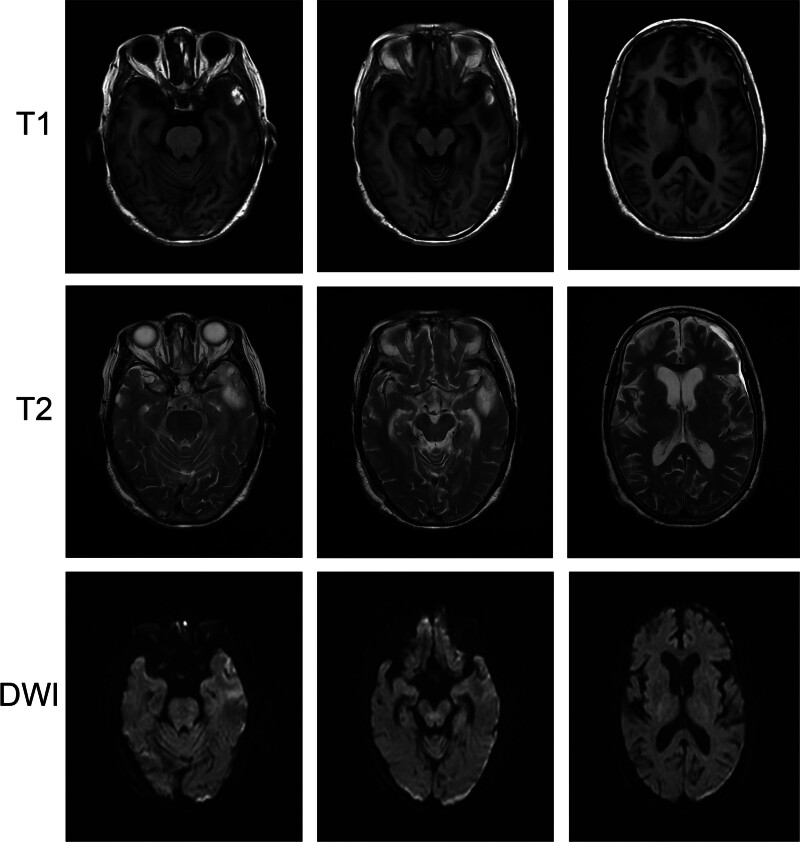
Head MRI image after considering Guillain–Barré syndrome. Left temporal band of long T1 and T2 signal shadows with high signal on DWI. DWI = diffusion-weighted imaging, MRI = magnetic resonance imaging.

The examination of head CT and magnetic resonance imaging showed no intracranial delayed hemorrhage and cerebral infarction, and Guillebalan syndrome was considered in Figures 4 and 5. The possibility of GBS was suggested and the lumbar puncture was subsequently performed.

Intravenous immunoglobulin (IVIG) 2 g/kg was given daily. Five days later, the symptoms improved significantly, including spontaneous breathing strength, limb muscle strength recovered to grade 2, pain and temperature sensation recovered, and physical rehabilitation was continued. Six weeks later, the patient was discharged with grade 4 muscle strength recovery. This further confirmed the diagnosis of GBS.

## 3. Discussion

In fact, the development of GBS after traumatic brain injury was first reported in 1987.^[10]^ Since then, there have been a few reports of GBS after traumatic brain injury in the past 3 decades. However, to date, this is the first report of GBS after posterior fossa decompression of severe traumatic brain injury accompanied by positive anti-sulfatide antibodies only in serum, and an interesting observation in our case is the asymmetric presentation of his symptoms. Clinically, GBS is usually defined by bilateral symmetrical paresis and hyporeflexia of the limbs, and starts in the distal lower extremities, but can start more proximally in the legs or arms.^[11]^ Logullo et al^[12]^ have been demonstrated in GCS and has been attributed to varying extents of immune-mediated pathology on either side of the sagittal plane. In addition, patients may present with cranial nerve involvement resulting in facial, oculomotor, or bulbar weakness, as in Miller Fisher syndrome, which might then extend to involve the.^[8]^

Although GBS is self-limited and immunotherapy has a certain effect, 5% of patients may die, and 20% of patients may be left with severe dysfunction.^[13]^ Studies have found that anti-glucolipid antibodies (including anti-ganglioside antibodies and anti-sulfatide antibodies) on the surface of peripheral nerve membranes are closely related to the pathogenesis of GBS.^[14]^ Sulfatide, also known as sulfatide, is an acidic glycolipid on myelin membrane that contains sulfuric acid residues and is different from ganglioside. It is very abundant in the nervous system, mainly located in the myelin sheath, and it plays an important role in maintaining the structure and physiological function of the nerve sheath.^[15]^ Under pathological conditions, sulfatide can promote the transverse growth of myelin sheath, affect its sorting and assembly, and affect the normal function of myelin proteins.^[16]^ Some scholars have made an animal model of peripheral neuropathy by sensitizing guinea pigs with sulfatide, and confirmed that sulfatide is involved in the occurrence and development of peripheral neuropathy.^[17]^ Moreover, some scholars believe that screening anti-sulfatide antibodies can be used as one of the basis for the diagnosis of peripheral neuropathy.^[18]^

At present, the pathogenic mechanism of anti-sulfatide antibodies in inflammatory peripheral neuropathy is not very clear. Some studies have found that patients with high titers of anti-sulfatide antibodies have widened myelin membrane gap under ultrastructure, and IgM-anti-sulfatide antibodies and complement factors are deposited on the myelin sheath of such patients by indirect immunofluorescence analysis, which may be the cause of peripheral nerve injury.^[19]^

Diagnosis of GBS is made based on symptoms and physical examination findings.^[1]^ The role of CSF testing and electrophysiological examination in the diagnosis of GBS is very important. Except for the case reported by Duncan and colleagues in 1987 without a lumbar puncture examination,^[10]^ all cases showed albuminocytological dissociation in CSF. The examination results of our case were consistent with the report. However, due to the rapid progression of the disease, the patient could not be weaned from the ventilator in the early stage, so the opportunity of neuroelectrophysiological examination was missed. Moreover, anti-sulfatide antibodies are closely associated with GBS.^[7]^ Some scholars have proposed that immunoglobulin trial treatment is also a way to diagnose GBS.^[20]^

GBS is an immune-mediated acute inflammatory peripheral neuropathy, so the main treatment at present is still immunotherapy.^[21]^ Intravenous IVIG and plasma exchange are the preferred treatment for GBS. IVIG regimen was 400 mg/(kg·d) by intravenous drip for 3 to 5 days. The plasma exchange protocol was 30 to 50 mL/kg, 3 to 5 times in 1 to 2 weeks.^[13]^ Although early studies have shown that plasma exchange is more likely to prevent the progression of GBS than IVIG, the incidence of adverse reactions is similar between plasma exchange and IVIG.^[22]^ However, IVIG is easier to administer clinically, so it is more widely used and usually preferred. Although some scholars have previously suggested that glucocorticoids can slow down the progression of the disease by reducing inflammation,^[23]^ 8 randomized controlled trials showed that corticosteroids had no significant effect on the treatment of GBS, and oral corticosteroids even brought varying degrees of adverse reactions.^[24]^ In addition, plasma exchange followed by IVIG was not superior to either monotherapy, and there was no significant difference in efficacy between IVIG combined with intravenous methylprednisolone and IVIG alone.^[24,25]^

## 4. Conclusion

GBS with positive anti-sulfatide antibody after craniotomy in adults is a very rare condition. It is difficult to diagnose because of its atypical clinical manifestations. However, clinicians should identify post-traumatic GBS when sudden limb weakness occurs in patients without limb dysfunction after severe traumatic brain injury. The detection of ganglioside antibody spectrum in CSF and plasma, neuroelectrophysiological examination, nerve biopsy, and routine detection of CSF are of guiding significance for the differential diagnosis of GBS. Timely and effective treatment in the acute stage of GBS is the key to reduce the mortality and disability rate, but the complete remission of symptoms may take a long time.

## Acknowledgments

The authors are grateful to the patient and his family for their support and cooperation.

## Author contributions

**Conceptualization:** Xiaobin Min, Haoye Feng, Hongjun Su.

**Data curation:** Xiaobin Min, Haoye Feng, Riguang Zhao, Zhigang Guo, Hongjun Su.

**Formal analysis:** Xiaobin Min, Haoye Feng, Riguang Zhao, Zhigang Guo, Hongjun Su.

**Visualization:** Xiaobin Min, Haoye Feng, Hongjun Su.

**Writing—original draft:** Xiaobin Min, Haoye Feng, Hongjun Su.

**Writing—review & editing:** Xiaobin Min, Haoye Feng, Hongjun Su.

**Funding acquisition:** Hongjun Su.

## References

[1] Marcus R. What is Guillain-Barré syndrome? JAMA. 2023;329:602. 10.1001/jama.2022.24232 36729424

[2] Fokke C van den Berg B Drenthen J Walgaard C van Doorn PA Jacobs BC. Diagnosis of Guillain-Barre syndrome and validation of Brighton criteria. Brain. 2014;137(Pt 1):33–43. 24163275 10.1093/brain/awt285

[3] Willison HJ Jacobs BC van Doorn PA. Guillain-Barre syndrome: surveillance and cost of treatment strategies—authors’ reply. Lancet. 2017;389:253–4. 10.1016/S0140-6736(17)30055-7 28118915

[4] Laman JD Huizinga R Boons GJ Jacobs BC. Guillain-Barre syndrome: expanding the concept of molecular mimicry. Trends Immunol. 2022;43:296–308. 35256276 10.1016/j.it.2022.02.003 PMC9016725

[5] Li HF Hu SM Lv M. Association of Ad26.COV2.S COVID-19 vaccine with presumptive Guillain-Barre syndrome. JAMA. 2022;327:392–3. 10.1001/jama.2021.23003 35076675

[6] Lleixa C Martin-Aguilar L Pascual-Goni E. Autoantibody screening in Guillain-Barre syndrome. J Neuroinflammation. 2021;18:251. 34719386 10.1186/s12974-021-02301-0 PMC8559393

[7] Liu J Tang F Chen X Li Z. Guillain-Barre Syndrome with incomplete oculomotor nerve palsy after traumatic brain injury: case report and literature review. Brain Sci. 2023;13:527. 37190493 10.3390/brainsci13040527 PMC10136930

[8] Willison HJ Jacobs BC van Doorn PA. Guillain-Barre syndrome. Lancet. 2016;388:717–27. 26948435 10.1016/S0140-6736(16)00339-1

[9] Hass RM Wijdicks EFM. Locked in from fulminant GBS after lumbar spine surgery. Pract Neurol. 2024;24:63–5. 37890999 10.1136/pn-2023-003925

[10] Duncan R Kennedy PG. Guillain-Barre syndrome following acute head trauma. Postgrad Med J. 1987;63:479–80. 3432176 10.1136/pgmj.63.740.479 PMC2428316

[11] Li X Zhang C. Guillain-Barré syndrome after surgery: a literature review. Front Neurol. 2024;15:1368706. 38638310 10.3389/fneur.2024.1368706 PMC11024248

[12] Logullo F Manicone M Di Bella P Provinciali L. Asymmetric Guillain-Barre syndrome. Neurol Sci. 2006;27:355–9. 17122947 10.1007/s10072-006-0710-z

[13] Leonhard SE Mandarakas MR Gondim FAA. Diagnosis and management of Guillain-Barre syndrome in ten steps. Nat Rev Neurol. 2019;15:671–83. 31541214 10.1038/s41582-019-0250-9 PMC6821638

[14] Wanleenuwat P Iwanowski P Kozubski W. Antiganglioside antibodies in neurological diseases. J Neurol Sci. 2020;408:116576. 31726381 10.1016/j.jns.2019.116576

[15] Palavicini JP Wang C Chen L. Novel molecular insights into the critical role of sulfatide in myelin maintenance/function. J Neurochem. 2016;139:40–54. 27417284 10.1111/jnc.13738 PMC5037006

[16] Campagnolo M Ferrari S Dalla Torre C. Polyneuropathy with anti-sulfatide and anti-MAG antibodies: clinical, neurophysiological, pathological features and response to treatment. J Neuroimmunol. 2015;281:1–4. 25867460 10.1016/j.jneuroim.2015.02.009

[17] Qin Z Guan Y. Experimental polyneuropathy produced in guinea-pigs immunized against sulfatide. Neuroreport. 1997;8:2867–70. 9376521 10.1097/00001756-199709080-00013

[18] Nobile-Orazio E Gallia F Terenghi F Allaria S Giannotta C Carpo M. How useful are anti-neural IgM antibodies in the diagnosis of chronic immune-mediated neuropathies? J Neurol Sci. 2008;266:156–63. 17915254 10.1016/j.jns.2007.09.020

[19] Ho TW Mishu B Li CY. Guillain-Barre syndrome in northern China. Relationship to *Campylobacter jejuni* infection and anti-glycolipid antibodies. Brain. 1995;118(Pt 3):597–605. 7600081 10.1093/brain/118.3.597

[20] Carr KR Shah M Garvin R Shakir A Jackson C. Post-traumatic brain injury (TBI) presenting with Guillain-Barre syndrome and elevated anti-ganglioside antibodies: a case report and review of the literature. Int J Neurosci. 2015;125:486–92. 25158009 10.3109/00207454.2014.957760

[21] Hagen KM Ousman SS. The neuroimmunology of Guillain-Barre syndrome and the potential role of an aging immune system. Front Aging Neurosci. 2020;12:613628. 33584245 10.3389/fnagi.2020.613628 PMC7873882

[22] Hughes RA Swan AV van Doorn PA. Intravenous immunoglobulin for Guillain-Barre syndrome. Cochrane Database Syst Rev. 2014;2014:CD002063. 11406030 10.1002/14651858.CD002063

[23] Berciano J. The rationale for the use of corticosteroids in early severe Guillain-Barre syndrome. Autoimmun Rev. 2021;20:102907. 34274541 10.1016/j.autrev.2021.102907

[24] Hughes RA Swan AV Raphael JC Annane D van Koningsveld R van Doorn PA. Immunotherapy for Guillain-Barre syndrome: a systematic review. Brain. 2007;130(Pt 9):2245–57. 17337484 10.1093/brain/awm004

[25] van Koningsveld R Schmitz PI Meche FG Visser LH Meulstee J van Doorn PA; Dutch GBS study group. Effect of methylprednisolone when added to standard treatment with intravenous immunoglobulin for Guillain-Barre syndrome: randomised trial. Lancet. 2004;363:192–6. 14738791 10.1016/s0140-6736(03)15324-x

